# Successful pharmaco-invasive approach using a lower alteplase dose and VA-ECMO support in high-risk pulmonary embolism: case report

**DOI:** 10.3389/fcvm.2024.1444636

**Published:** 2024-07-22

**Authors:** Paola Gutierrez-Gallegos, Vicente Jimenez-Franco, Carlos Jerjes-Sanchez, Renata Quevedo-Salazar, Jahir Rodriguez-Rivera, Enrique Paredes-Gutierrez, Daniel Lira-Lozano, Juan Quintanilla-Gutierrez, Rene Gomez-Gutierrez, Erasmo de la Peña-Almaguer, Guillermo Torre-Amione

**Affiliations:** ^1^ Tecnologico de Monterrey, Escuela de Medicina y Ciencias de la Salud, Monterrey, Mexico; ^2^ Instituto de Cardiología y Medicina Vascular, TecSalud, San Pedro Garza Garcia, Mexico; ^3^ Shock Team, Instituto de Cardiología y Medicina Vascular, TecSalud, San Pedro Garza Garcia, Mexico

**Keywords:** high-risk pulmonary embolism, cardiac arrest, thrombolysis, extracorporeal life-support, ECMO

## Abstract

Despite the elevated mortality rates associated with high-risk pulmonary embolism (PE), this condition remains understudied. Data regarding the effectiveness and safety of invasive therapies such as venoarterial extracorporeal membrane oxygenation (VA-ECMO) in this patient population remains controversial. Here, we present the case of a 61-year-old male with high-risk PE associated with refractory cardiac arrest and cardiogenic shock who underwent a combination of extracorporeal cardiopulmonary resuscitation with VA-ECMO and pharmaco-invasive therapy (mechanical thrombi fragmentation plus lower alteplase dose), resulting in successful pulmonary reperfusion. After a prolonged in-hospital stay, the patient was discharged in stable condition.

## Introduction

Pulmonary embolism (PE) remains the third leading cause of cardiovascular death worldwide, behind myocardial infarction and stroke (1). Despite improvements in advanced treatments, high-risk PE continues with high mortality rates ranging from 15% to 80% (2). Mechanical circulatory support, specifically venoarterial extracorporeal membrane oxygenation (VA-ECMO), has been increasingly used in high-risk PE associated with refractory or resuscitated cardiac arrest, refractory cardiogenic shock (CS), and failed thrombolysis (1). On the one hand, current evidence suggests that VA-ECMO use in this population may not yield a significant improvement in mortality and, conversely, may increase the risk of major bleeding complications (1, 3). On the other hand, alternative evidence shows that early use of VA-ECMO may result in up to a 95% survival rate (1, 2). Moreover, the effectiveness and safety of advanced treatments with VA-ECMO in high-risk PE patients remain unclear (1–3). Here, we present the case of a 61-year-old male with high-risk PE associated with cardiac arrest and SCAI E CS. He was supported by VA-ECMO and underwent a pharmaco-invasive approach using a lower alteplase dose, resulting in successful pulmonary reperfusion.

## Case presentation

**Figure 1:**
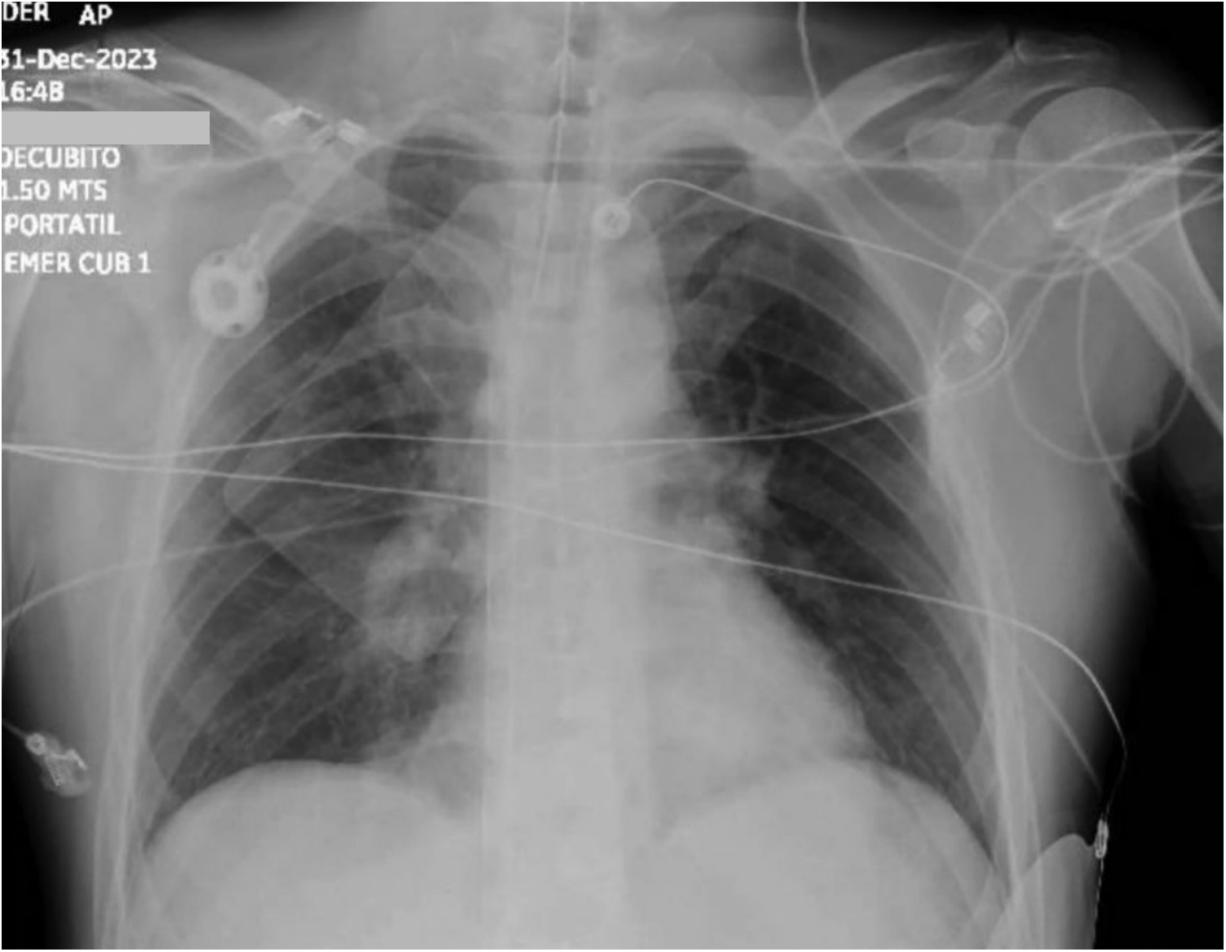
Chest x-ray shows right pulmonary artery amputation and bilateral Westermark sign.

On December 31, a 61-year-old male presented to the emergency room with a 30-min history of sudden-onset resting dyspnea associated with diaphoresis, cyanosis, and weakness. His past medical history included a recent diagnosis of colon cancer treated with colectomy and oxaliplatin-based chemotherapy. On arrival, the patient's temperature was 35.6°C. He was clinically unstable, with hypotension (52/42 mmHg), bradycardia (41 bpm), tachypnea (26 rpm), and oxygen desaturation (86%). On physical examination, chest auscultation revealed diminished cardiac sounds and bibasilar crackles, along with decreased respiratory sounds. Peripheral pulses were reduced, and the capillary refill time was 4 s. Ten minutes after admission, the patient underwent sudden cardiac arrest. Therefore, we initiated mechanical cardiopulmonary resuscitation (CPR) using a Lund University Cardiopulmonary Assist System (LUCAS) device with simultaneous activation of the shock team. We carried out eight cycles while concurrently administering 5 mg of epinephrine and 80 mEq of sodium bicarbonate. The patient returned to spontaneous circulation after 21 min of mechanical CPR. Initial venous blood gas analysis revealed pH <6.8, PO2 32 mmHg, PCO2 100 mmHg, and lactic acid >15 mmol/L. Initial chest x-ray during “code blue” activation showed right pulmonary artery amputation and bilateral Westermark sign (Figure 1). Subsequently, a second cardiac arrest occurred, requiring LUCAS-assisted CPR and five defibrillation events. The shock team concluded that conventional CPR had been unsuccessful and decided to initiate VA-ECMO while continuing chest compressions (extracorporeal CPR). Using ultrasound guidance, a 5 French micropuncture kit was employed to access the common femoral artery and vein. Compressions were paused for up to 60 s during vessel needling, with the team leader overseeing these intervals. A 0.035-inch venous guidewire was confirmed to be in the inferior vena cava, and the arterial guidewire was then advanced. We used the arterial and venous introducer cannulas for vessel dilation instead of serial dilators, as this method was safer and reduced cannulation time. A femoral artery distal perfusion cannula was not required for the initial deployment. We also started unfractionated heparin, aiming for an activated thromboplastin time between 180 and 220 s. Laboratory results revealed a D-Dimer (DD) 38,892 mcg/ml (0–500 mcg/ml), high sensitivity troponin (hs-c-TnI) 7 ng/L (0–35 ng/L), and B-type natriuretic peptide (BNP) 106 pg/ml (<100 pg/ml). Cardiac tracking with point-of-care ultrasound showed right atrial and ventricular dilation with interventricular septum deviation and left ventricular collapse. An ECG showed sinus rhythm, flattened T waves in the right precordial (V1) and inferior (II, III and aVF) leads, and ST depression from V4 to V6. We established a high clinical suspicion of high-risk PE associated with recurrent cardiac arrest and SCAI E CS based on risk factors, clinical presentation, imaging findings, and biomarkers. CT angiography was not performed due to the patient's unstable condition.

**Figure 2:**
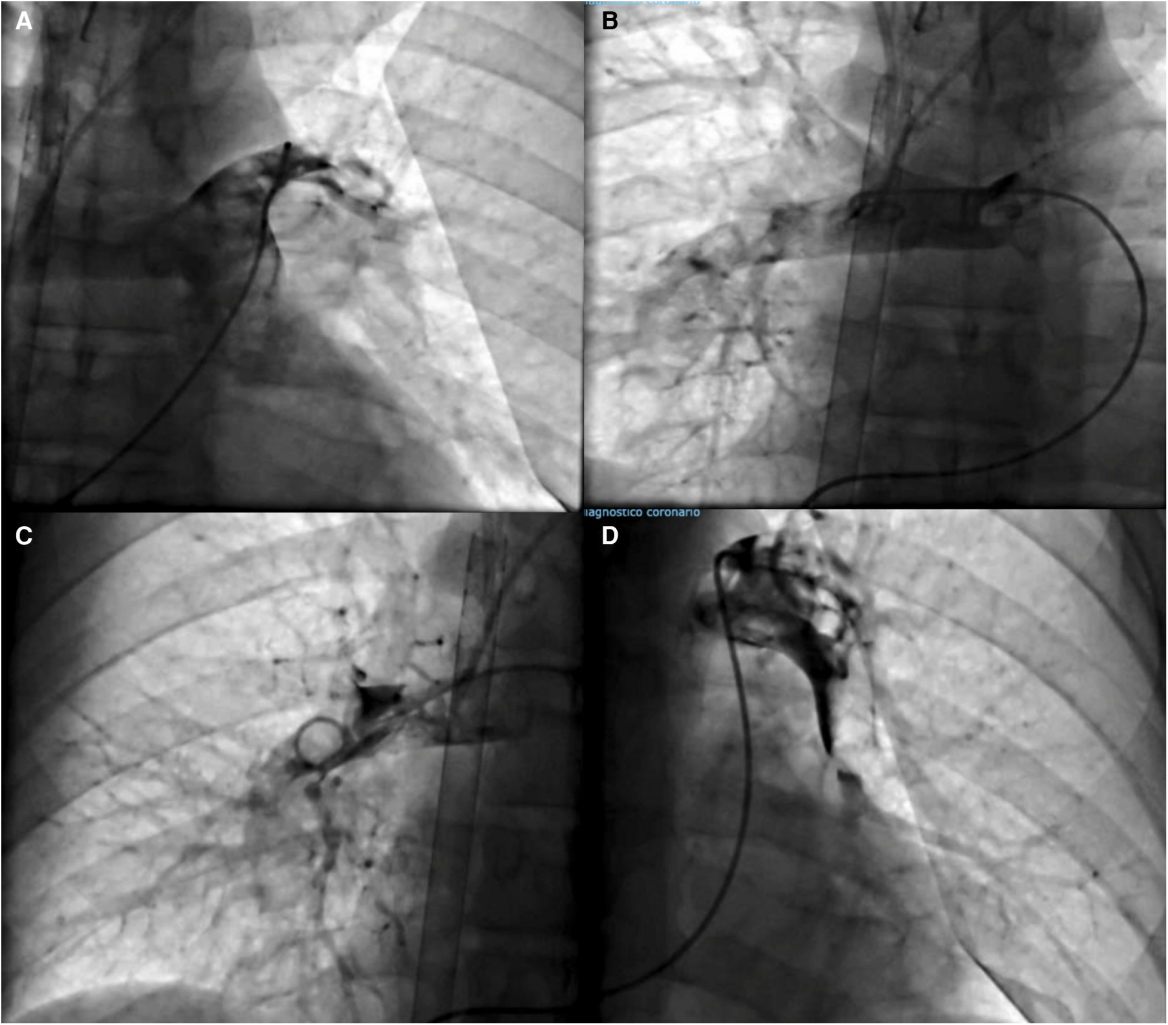
(**A**, **B**) Pulmonary angiography shows a filling defect in both principal pulmonary arteries. (**C**, **D**) Final angiography showing improved pulmonary circulation after pharmacoinvasive treatment.

**Figure 3:**
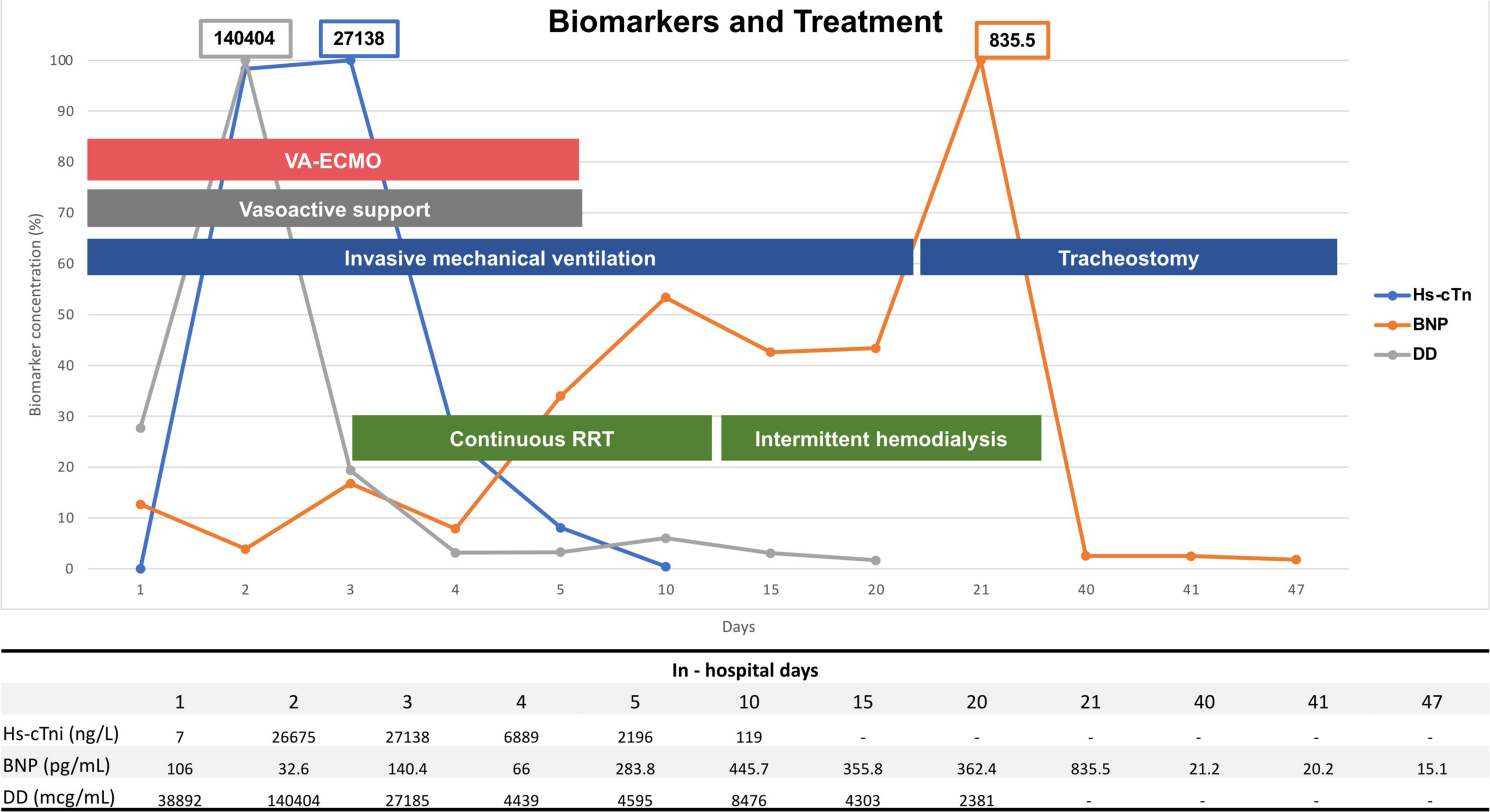
Biomarkers on admission and during in-hospital follow-up. Main invasive treatment strategies are also shown. DD gray line, BNP orange line, Hs-cTni blue line. DD, D-Dimer; BNP, B-type natriuretic peptide; Hs-cTni, high sensitivity troponin I; RRT, renal replacement therapy; VA-ECMO; venoarterial extracorporeal membrane oxygenation.

The patient was subsequently transferred to the catheter lab. We used a left femoral approach employing a 6 French pigtail catheter. Pulmonary angiography showed a high thrombus burden in both principal pulmonary arteries (Figure 2). We initiated a pharmaco-invasive approach involving manual mechanical fragmentation followed by catheter-directed thrombolysis using 5 mg of alteplase in each pulmonary artery, markedly improving pulmonary circulation (Figure 2). Coronary angiography excluded obstructive coronary disease. After successful pharmaco-invasive therapy, the patient was admitted to the ICU. On the following day, the patient remained under VA-ECMO support, requiring vasopressor infusion with norepinephrine, vasopressin, and milrinone due to hemodynamic instability and low pulsatility. A transthoracic echocardiogram showed right ventricular dilatation (43 mm), mild global hypokinesis, reduced biventricular systolic function with a left ventricular ejection fraction (LVEF) of 46%, and tricuspid annular plane systolic excursion (TAPSE) of 14 mm. Follow-up laboratories showed DD 140,404 mcg/ml, hs-c-TnI 26,675 ng/L, BNP 32.6 pg/ml, pH 7.33, PCO2 55 mmHg, and lactate 9.4 mmol/L (Figure 3). On the third day, creatinine levels increased to 2.8 mg/dl, along with a decreased urine output, prompting the initiation of continuous renal replacement therapy.

By the fourth day, vasopressors and inotropes were weaned off. On the fifth day, VA-ECMO was decannulated after progressively reducing flow rates to 1.5 L/min and ensuring stable hemodynamics. The venous cannula was removed first, followed by the arterial cannula. A Viabahn endoprosthesis (WL Gore & Associates, Flagstaff, Ariz) was then placed, achieving complete percutaneous closure without complications. A control pulmonary angiography revealed a remarkable improvement in pulmonary circulation. Right heart catheterization showed a right atrial pressure of 8 mm Hg, a mean pulmonary artery pressure of 26 mmHg, a pulmonary capillary wedge pressure of 18 mmHg, a cardiac output of 5.2 L/min, a cardiac index of 3.07 L/min/m^2^, a pulmonary artery pulsatility index (PAPi) of 2.5 and a cardiac power output (CPO) of 1.13 W. On day seven, the patient had an episode of atrial fibrillation that reverted to sinus rhythm with pharmacologic cardioversion. Additionally, 1,200 ml of serosanguineous pleural fluid was drained via thoracentesis, likely resulting from pulmonary contusion due to chest compressions, an infectious process or severe right heart failure.

**Figure 4:**
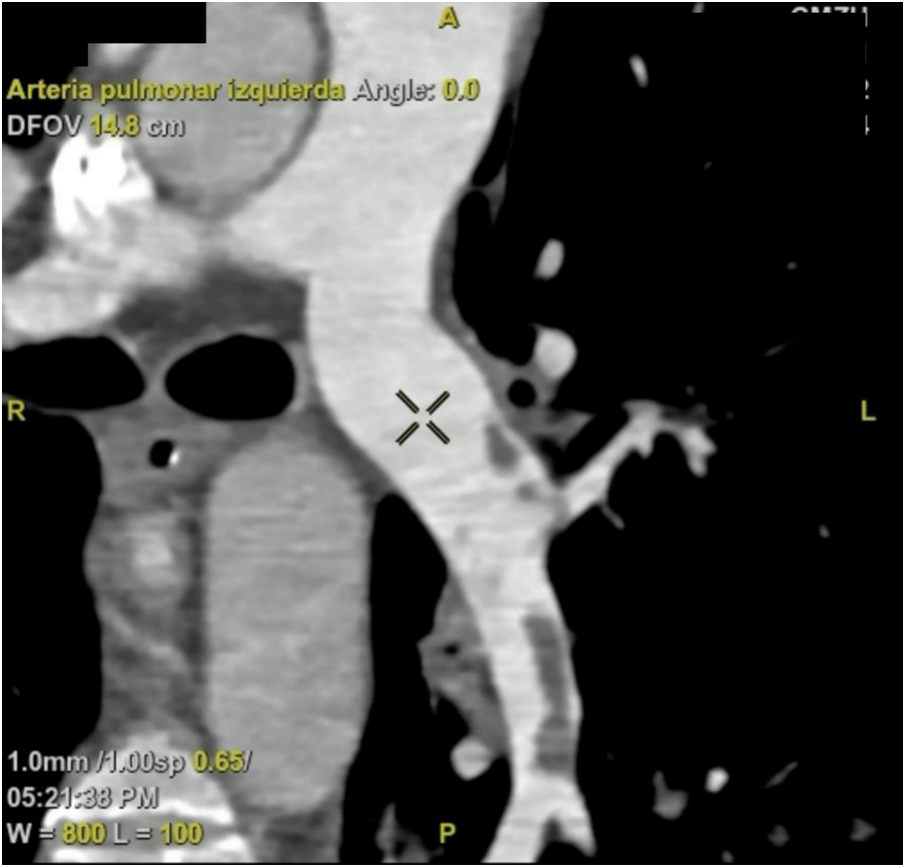
CT angiography. The left pulmonary artery exhibits a partial filling defect in the segmental and subsegmental branches. CT, computed tomography.

On day nine, the patient developed gram-negative pneumonia due to E. cloacae and K. pneumonia. Additionally, we switched unfractioned heparin to enoxaparin 80 mg and started intermittent hemodialysis. On the 14th day, a follow-up pulmonary CT angiography showed partial filling defects at the left pulmonary artery's main bifurcation, extending towards its segmental and subsegmental branches (Figure 4). On day 15, we started metoprolol 95 mg due to recurrent episodes of atrial fibrillation with rapid ventricular response. On the 20th day, following two unsuccessful extubation attempts, a tracheostomy was performed. On day 23, upon sedation withdrawal, we identified paraplegia, which was attributed to prolonged cardiac arrest. The neurological examination of the lower limbs showed no voluntary movement, absent deep tendon reflexes, and reduced sensation to pain and temperature with preserved proprioception and vibration. Thoracic and lumbar spine magnetic resonance imaging revealed diffuse myelitis and ischemia of the anterior horn of the spinal cord. On the 27th day, an echocardiogram showed an improved LVEF of 54%, a TAPSE of 26 mm, and absence of right ventricular dilation (31 mm).

The patient was then transferred to the inpatient floor, and physical therapy was started. On day 47, the patient was discharged in stable condition on apixaban 5 mg twice daily and metoprolol 95 mg daily. Despite the improved neurological condition, paraplegia remained.

## Discussion

In this case, the key findings were as follows: First, the shock team and VA-ECMO support played a pivotal role as a salvage therapy for the patient's critical condition. Second, we performed a successful pharmaco-invasive approach involving mechanical thrombi fragmentation followed by catheter-directed thrombolysis using a lower alteplase dose without major bleeding complications. Third, despite severe right ventricular dysfunction and CS, the patient's BNP and hs-c-TnI measurements were discordantly low upon admission, likely due to the time interval between symptom onset and the collection of the initial biomarker samples. Fourth, the utility of point-of-care ultrasound in the diagnosis of acute PE. Finally, the initially reduced LVEF and subsequent recovery suggest the involvement of ventricular interdependence in the setting of severe right ventricular dysfunction.

Current evidence suggests that PE mortality rates have increased over the last decade despite technological advances in PE care and the increasing adoption of novel healthcare delivery models for these patients, such as using multidisciplinary PE response teams (4). The high-risk PE phenotype represents severely ill patients, with short-term mortality rates previously estimated between 15% and 80% (2). Despite the high mortality rates, most clinical research is focused on intermediate-risk PE. Consequently, the hemodynamic profile and the optimal advanced treatment in this understudied population are not well-defined (4).

To date, ECMO has emerged as a promising treatment modality for high-risk PE associated with refractory or resuscitated cardiac arrest, refractory CS, and failed thrombolysis (5). The rationale for VA-ECMO support in right ventricular failure is to redirect blood from the right atrium to the arterial circulation, thus reducing right ventricular overload, restoring ventricular interdependence, and increasing left ventricular output (6, 7). A retrospective study by Pasrija et al. demonstrated a 95% survival rate in patients with high-risk PE and end-organ dysfunction with early and aggressive use of VA-ECMO (2). However, evidence from a national database showed that ECMO use had no difference in mortality compared to standard treatment strategies and was associated with a higher incidence of in-hospital complications in 820 high-risk PE patients (1). Additionally, a retrospective observational study of the Extracorporeal Life Support Organization database revealed an in-hospital mortality rate of 68.1% among 821 patients undergoing extracorporeal CPR (3). As a result, the efficacy and safety of VA-ECMO in high-risk PE patients remain uncertain. In our case, VA-ECMO was successfully employed to achieve clinical stability before implementing the pharmaco-invasive approach (5). This practice was aligned with the ESC Working Group on Pulmonary Circulation and Right Ventricular Function recommendations, which support ECMO use in refractory circulatory collapse or cardiac arrest as a bridge to advanced treatment (IIb) (8, 9). Although ECMO use is strongly associated with bleeding complications, our patient's age, absence of cardiovascular comorbidities, early ECMO discontinuation, and the shock team's experience could explain the lack of bleeding events.

Meanwhile, the American College of Chest Physicians' clinical practice guidelines (10) suggest catheter-directed thrombolysis for high-risk PE patients associated with increased bleeding risk, failed systemic thrombolysis, or shock likely to cause death before systemic thrombolysis can take effect (5). Nonetheless, limited data exist on catheter-directed thrombolysis alongside ECMO, with only four published studies showing a 25% mortality rate in 16 patients treated with ultrasound-facilitated catheter-directed thrombolysis (5). In our case, the clinical decision to pursue a pharmaco-invasive approach with mechanical thrombi fragmentation followed by catheter-directed thrombolysis using alteplase 5 mg in each main pulmonary artery was encouraged by previous Mexican experience (11), in which patients underwent successful thrombus fragmentation with a pigtail catheter, followed by thrombus aspiration and alteplase 10 mg. We decided to use alteplase 5 mg based on the results of the OPTALYSE study, where 4 mg per lung using the EKO system was safe and effective (12). The combination of right ventricular unloading with VA-ECMO and pharmaco-invasive therapy significantly improved right ventricular function in our patient, as evidenced by echocardiographic findings.

BNP and hs-c-TnI are valuable biomarkers in patients with high clinical suspicion of PE. It has been suggested that in patients presenting to the emergency room with BNP measurements <100 pg/ml, right ventricular dysfunction is unlikely, while increased values have been correlated with a 10% risk of early death and a 23% risk of adverse clinical outcomes (13). Our patient's discordantly low BNP levels (106 pg/ml) upon admission might be attributed to the short interval between symptom onset and the first sample collection (30 min). Considering that the BNP's half-life is 23 min, approximately 2 h are required to reflect changes in the right ventricular function (16). Hence, a BNP measured within the first 60 min of acute right ventricular dysfunction will likely have low diagnostic accuracy.

Similarly, discordantly low BNP levels upon admission have been observed in conditions such as flash pulmonary edema, acute pulmonary edema secondary to papillary muscle rupture, right ventricular myocarditis secondary to systemic lupus erythematosus, and intermediate high-risk pulmonary embolism, all of which present with an acute onset of less than 2 h (13). Emergency medicine physicians, as frontline responders to patients with acute ventricular dysfunction, should be aware of this 2-h lag to avoid underdiagnosis. In our case, BNP measurements remained below 106 pg/ml in the first four days, indicating the effectiveness of the pharmaco-invasive approach (Figure 2) and right ventricular function improvement. Nonetheless, in the subsequent days, BNP values increased, possibly secondary to acute renal injury.

On the other hand, hs-c-TnI expression involves multifactorial mechanisms. These include physiologic processes such as myocyte cell turnover, increased cellular wall permeability, the release of troponin degradation products, and the active secretion of vesicles -and pathological processes- such as ischemia, necrosis, apoptosis, and necroptosis (14, 15). Despite our patient's severe right ventricular dysfunction, their hs-c-TnI measurements were within the normal range (7 ng/L). This could be explained by the kinetics of cardiac troponins in myocardial infarction, where hs-c-TnI slightly increases initially, indicating either an ischemia-induced release of the “early-release pool” or micronecrosis, followed by a significant increase within 2–6 h, reflecting extensive myocardial necrosis and degradation of myofibrillar proteins (16). Nevertheless, previous evidence has demonstrated that in patients with fulminant myocarditis, normal or mildly increased cardiac troponin measurements upon admission are associated with worse in-hospital and mid-term outcomes (17). Further, among patients with non-ST-elevation myocardial infarction, low-level elevations in hs-c-TnT were associated with a threefold increase in short-term risk for cardiovascular death or recurrent myocardial infarction (18), suggesting treatment should be pursued despite low troponin levels. In our patient, the significant elevation of hs-c-TnI in the subsequent days suggests a type 2 myocardial infarction (15).

Another important aspect to highlight is the role of point-of-care ultrasound in the diagnosis of acute pulmonary embolism. Our patient experienced cardiac arrest 10 min after arriving at the emergency room, reflecting the severity of his condition. In these time-sensitive situations, the utility of echocardiography in identifying reversible causes of cardiac arrest has been well-documented (19–21). In fact, the European Society of Cardiology (ESC) guidelines recommend using cardiac and venous ultrasound for patients admitted to the ER with hemodynamic instability and suspected PE (9). Additionally, further studies suggest complementing with lung ultrasound in the so-called “triple ultrasound” approach (22–24). Some of the findings suggestive of PE include right ventricular dilation, interventricular septal deviation and restricted left ventricular filling (19–21, 25), all of which were present in our patient. Conversely, in the absence of signs of right ventricular dysfunction, PE can be ruled out (23, 26). Limitations worth mentioning include the need for specific operator training, the potential to miss important details due to time restrictions, and the overlapping findings between differential diagnoses (23, 27).

An unexpected finding in our case was the initially reduced LVEF (46%). In PE with a high thrombus burden, increased pulmonary vascular resistance arises from direct flow impedance, local hypoxic vasoconstriction, and platelet-thrombin-induced vasoactive peptide release, leading to increased right ventricular afterload (28). This results in increased right ventricular wall tension, ischemia, and septal deviation, which directly impact left ventricular dimensions and pressures. Ultimately, this can lead to cardiogenic shock and potentially cardiac arrest (29–33) (which is often preceded by bradycardia, as seen in our case). This suggests that ventricular interdependence plays a role in severe right ventricular dysfunction and could explain the patient's initial slight decrease in LVEF, which recovered to 54% during follow-up. Substantial evidence is currently lacking to fully understand left ventricular performance in severe right ventricular dysfunction among high-risk PE patients.

Finally, our patient's neurological outcome was attributed to recurrent and refractory cardiac arrest. The spinal cord's blood supply is highly vulnerable to ischemia, especially in the setting of low cardiac output and hypoperfusion (34), as seen in prolonged cardiac arrest. Additionally, CPR poses the risk of dislodging thrombi, directly impeding blood flow (35). However, it is crucial to consider other possible, albeit less probable causes, such as the “watershed phenomenon,” where there's competition between the ECMO circuit's oxygenated blood and native cardiac output (34), which has an incidence of approximately 0.3% according to the largest case series reported (36). Potential management strategies, such as altering cannulation site, increasing perfusion pressure, spinal drainage and inferior vena cava filters have been used in similar scenarios; however, their effectiveness in this specific setting remains unknown (34, 37, 38).

## Conclusion

Our case illustrates the complexity of clinical decision-making in high-risk PE patients, particularly those associated with refractory cardiac arrest and CS supported by ECMO. Although VA-ECMO has emerged as a promising therapy, its effectiveness in PE remains controversial, and randomized controlled trials are needed to evaluate its efficacy and safety. In our case, the combination of ECMO and pharmaco-invasive therapy using a lower alteplase dose achieved pulmonary reperfusion without major bleeding complications. However, the optimal advanced treatment in this patient population remains unclear. Insights from this case may aid similar challenging scenarios in clinical decision-making, ultimately improving patient care.

## Data Availability

The original contributions presented in the study are included in the article/Supplementary Material, further inquiries can be directed to the corresponding author.
